# System-Based Interventions to Address Physician Burnout: A Qualitative Study of Canadian Family Physicians’ Experiences During the COVID-19 Pandemic

**DOI:** 10.34172/ijhpm.8166

**Published:** 2024-06-19

**Authors:** Maria Mathews, Samina Idrees, Dana Ryan, Lindsay Hedden, Julia Lukewich, Emily Gard Marshall, Judith Belle Brown, Paul Gill, Madeleine McKay, Eric Wong, Leslie Meredith, Lauren Moritz, Sarah Spencer

**Affiliations:** ^1^Department of Family Medicine, Schulich School of Medicine & Dentistry, Western University, London, ON, Canada.; ^2^Faculty of Nursing, Memorial University, St. John’s, NL, Canada.; ^3^Faculty of Health Sciences, Simon Fraser University, Burnaby, BC, Canada.; ^4^Department of Family Medicine Primary Care Research Unit, Dalhousie University, Halifax, NS, Canada.; ^5^Doctors Nova Scotia, Dartmouth, NS, Canada.

**Keywords:** COVID-19, Family Physician, Burnout, Primary Care, Canada, Qualitative Research

## Abstract

**Background:**

Medical professionals experienced high rates of burnout and moral distress during the COVID-19 pandemic. In Canada, burnout has been linked to a growing number of family physicians (FPs) leaving the workforce, increasing the number of patients without access to a regular doctor. This study explores the different factors that impacted FPs’ experience with burnout and moral distress during the pandemic, with the goal of identifying system-based interventions aimed at supporting FP well-being and improving retention.

**Methods:**

We conducted semi-structured qualitative interviews with FPs across four health regions in Canada. Participants were asked about the roles they assumed during different stages of the pandemic, and they were also encouraged to describe their well-being, including relevant supports and barriers. We used thematic analysis to examine themes relating to FP mental health and well-being.

**Results:**

We interviewed 68 FPs across the four health regions. We identified two overarching themes related to moral distress and burnout: (1) inability to provide appropriate care, and (2) system-related stressors and buffers of burnout. FPs expressed concern about the quality of care their patients were able to receive during the pandemic, citing instances where pandemic restrictions limited their ability to access critical preventative and diagnostic services. Participants also described four factors that alleviated or exacerbated feelings of burnout, including: (1) workload, (2) payment model, (3) locum coverage, and (4) team and peer support.

**Conclusion:**

The COVID-19 pandemic limited FPs’ ability to provide quality care to patients, and contributed to increased moral distress and burnout. These findings highlight the importance of implementing system-wide interventions to improve FP well-being during public health emergencies. These could include the expansion of interprofessional team-based models of care, alternate remuneration models for primary care (ie, non-fee-for-service), organized locum programs, and the availability of short-term insurance programs to cover fixed practice operating costs.

## Background

**Key Messages**

**Implications for policy makers**

- While previous primary care reforms in Canada were designed to improve the quality of care provision in Canada, this study highlights the need for reforms to specifically address family physician (FP) burnout.
- This study proposes a number of system-based interventions for primary care, including an expansion of interprofessional team-based models of care, group practices, and the implementation of alternate payment models (ie, non-fee-for-service).
- These findings also highlight the need for short-term disability and illness insurance, as well as access to locum tenens programs, to assist FPs operating in fee-for-service practice models.
- Poor system-wide planning contributed to FP burnout during the COVID-19 pandemic; this study advocates for improved pandemic planning to alleviate workload issues related to the provision of care during future public health emergencies.

**Implications for the public**

In Canada, burnout has been attributed to a growing number of family physicians (FPs) leaving the workforce or narrowing their scope of practice, leading to an increase in the number of patients without a regular provider, a situation compounded by the COVID-19 pandemic. Additionally, burnout experienced on a professional level has been linked with disengagement and inefficiency, which could contribute to lower quality patient care. Given the existing challenges confronting the Canadian healthcare system, primary care reforms specifically designed to address FP well-being are needed. This study describes different factors that alleviated or exacerbated FP feelings of burnout and suggests concrete interventions to address these factors. These reforms have the potential to improve health outcomes, access to care, care coordination, and increase patient satisfaction. Through the implementation of these reforms, the healthcare system will be better positioned to recruit and retain FPs, resulting in a more sustainable, accessible, and equitable healthcare workforce.

Burnout is described as a “combination of overextension, disengagement and inefficacy”^1^ in combination with “repeated or lasting moral distress, the psychological phenomena whereby an individual feels unable to pursue what they believe to be an ethically appropriate course of action, due to systemic or institutionalized barriers.”^2-4^ Severe burnout may lead to a desire to leave a position or the profession.^5-9^ Burnout can be attributed to a number of causes, such as increased workload, a lack of work-life balance, rapidly changing policies/processes, long waitlists, and a lack of human resources.^10,11^ A national survey of physician health conducted by the Canadian Medical Association found that burnout is significantly higher among family physicians (FPs) compared with physicians practicing in other positions due to the higher prevalence of many of these factors in family medicine.^12^ In Canada, burnout has been attributed to the growing numbers of FPs who are leaving the workforce or narrowing their scope of practice, exacerbating the number of patients without a regular physician.^13^ Studies have reported high rates of burnout, anxiety, and stress among FPs in Canada^14-16^ and internationally^17-25^ prior to and as a result of the COVID-19 pandemic.^12,26-29^ National survey data also reports that 21% of FPs had felt morally distressed “very often” or “always” since the beginning of the pandemic, and 57% experienced burnout.^12^

 In Canada, provincial governments are responsible for the organization and delivery of health services, including primary care. FPs, who provide most primary care services, are largely independent, small business owners or sub-contractors. Over the last two decades, individual provinces have introduced voluntary, incremental primary care reforms that have resulted in each province having a myriad of primary care payment and practice models.^30-32^ Reforms, including the implementation of interprofessional teams, physician networks and group practices, and alternate forms of FP payment models (ie, non-fee-for-service), were generally introduced with the primary goal of improving the quality of care provided to patients.^29,30^ With the growing recognition of physician burnout, researchers increasingly have been calling for additional system reforms that specifically address the root causes of burnout within primary care, as this has been largely overlooked in the creation of previous reform policies.^6,14^ We examine factors that aggravated or alleviated FPs’ moral distress and burnout during the COVID-19 pandemic to identify system-based interventions to improve FP well-being.

## Methods

 This paper is part of a larger project examining FP experiences during the COVID-19 pandemic in Canada. A detailed overview of the study methods has been described previously.^33^ Briefly, we conducted semi-structured qualitative interviews with FPs in four health regions in Canada. Individuals were eligible to participate if they held a license to practice family medicine, as of the year 2020, in one of the following regions: the Vancouver Coastal health region in British Columbia, the Eastern Health region of Newfoundland and Labrador, the province of Nova Scotia (which has only one health region), or the Ontario Health West region. Recruitment and data collection occurred between the months of October 2020 and June 2021. We included physicians who practiced in traditional community-based health clinics, as well as long-term care homes or hospitals. Individuals completing residency training, on temporary licenses, or serving in only non-clinical roles (eg, academic, research, or administrative) were excluded.

 We used maximum variation sampling^34^ to ensure diversity across a wide range of sample characteristics, including participant gender, funding and practice model (eg, fee-for-service, alternative payment plans, etc), academic and hospital affiliation, and practice location (eg, rural, urban, etc). Research assistants in each health region identified potential participants using various online sources, including faculty lists, regional family practice lists, privileging lists, and physician search portals managed by provincial medical regulators. The identified physicians were invited to participate via email and were provided with relevant study information. We also promoted our study through postings in medical organizations’ newsletters and on social media. Additionally, we made use of snowball sampling in the health regions where it was permitted. Recruitment proceeded until the data reached saturation, defined as the point at which additional interviews no longer provided value-added insights.^34,35^ The data saturation point was determined through consultation with members of the research team.

 In each interview, participants were asked about the pandemic-related roles they assumed during different stages of the pandemic and the factors that impacted their ability to fulfil these roles. We did not ask specifically about moral distress or burnout, but encouraged participants to describe their well-being (including their mental health) and identify supports and barriers through probes. We also collected information on relevant demographic and practice characteristics, including participant gender, years in practice, work settings, clinic roles, practice location, and populations served. The interview guide was modified in each province to account for regional differences in health systems, pandemic response, and physician roles and responsibilities. Participants were provided with the opportunity to complete the interview via Zoom (Zoom Video Communications Inc) or by telephone. The interviews were audio-recorded, transcribed verbatim, and accompanied by interviewer field notes to aid in data analysis.

 We analyzed the data using thematic analysis. The initial coding of interviews occurred regionally, with each province having two members of the research team independently review two to three transcripts, identifying key ideas and forming a preliminary coding template. Each regional team then used their template to code a set of four interview transcripts (one from each province). In subsequent cross-provincial meetings, we compared coding and refined label definitions and descriptions to form a unified coding template, with conflicts being resolved through discussion and consensus. The unified template was then used across regions to code all transcripts and field notes. We coded the data using NVivo 12 (QSR International), a software designed to aid in qualitative research analysis. We summarized data on participant and practice characteristics using descriptive statistics.

 We took several steps to increase the robustness of our methodological approach and analysis,^34-36^ including pre-testing interview questions, documenting procedures, using experienced interviewers, and verifying meaning with participants in interviews. We made efforts to identify negative cases and provided rich descriptions and illustrative quotes. The public health leaders and FPs on our research team also played a key role in providing important practical expertise, informing the development of the interview guide, and assisting with the interpretation of findings.^37^

 This study was approved by the research ethics boards at Simon Fraser University, the University of British Columbia, the Health Research Ethics Board of Newfoundland and Labrador, Nova Scotia Health, and Western University. Participants provided informed consent prior to the scheduling of interviews. We maintained participant confidentiality through secure storage of audio-recordings, password-protection of electronic documents, and the de-identification of interview data in the transcription process.

## Results

 We interviewed a total of 68 FPs across the four regions (Table). The sample included 41 women, 46 FPs who were paid through alternate payment plans (ie, non-fee-for-service), and 20 FPs who worked in a rural community. This paper examines themes related to FP mental health, including formal and informal supports. These themes were discussed to varying degrees by all participants across all four regions. Participants described two overarching themes related to moral distress and burnout: (1) inability to provide appropriate care, and (2) and system-related stressors and buffers of burnout.

**Table:** Characteristics of Study Participants

	**Ontario ** **(n = 20)** **No. (%)**	**Nova Scotia ** **(n = 21)** **No. (%)**	**British Columbia ** **(n = 15)** **No. (%)**	**Newfoundland and Labrador (n = 12)** **No. (%)**	**Total ** **(n = 68)** **No. (%)**
Gender^a^					
Man	10 (50.0)	9 (42.9)	4 (26.7)	4 (33.3)	27 (39.7)
Woman	10 (50.0)	12 (57.1)	11 (73.3)	8 (66.7)	41 (60.3)
Practice type					
Fee-for-service	4 (20.0)	7 (33.3)	6 (40.0)	5 (41.7)	22 (32.4)
Alternative payment plan^b^	16 (80.0)	14 (66.7)	9 (60.0)	7 (58.3)	46 (67.6)
Hospital affiliation					
No	5 (25.0)	6 (28.6)	3 (20.0)	5 (41.7)	19 (27.9)
Yes	15 (75.0)	15 (71.4)	12 (80.0)	7 (58.3)	49 (72.1)
Community size^c^					
Rural	9 (45.0)	8 (38.1)	0 (0.0)	3 (25.0)	20 (29.4)
Small urban	1 (5.0)	0 (0.0)	0 (0.0)	0 (0.0)	1 (1.5)
Urban	8 (40.0)	13 (61.9)	15 (100.0)	8 (66.7)	44 (64.7)
Mix	2 (10.0)	0 (0.0)	0 (0.0)	1 (8.3)	3 (4.4)
Years in practice (mean)	18.7	15.4	16.9	16.3	16.9

^a^ Gender was asked as an open-ended question. ^b^ Alternate payment includes all non-fee-for-service or enhanced fee-for-service payment types. ^c^ Rural <10 000 population, Small urban =10 000-99 999 population, Urban >100 0000.

###  Inability to Provide Appropriate Care

 Participants expressed their concerns about the quality of care that their patients were able to receive during the COVID-19 pandemic, often citing instances where the care provided did not align with clinical guidelines and professional standards. They expressed anxiety about the fact that many patients were not able to access preventative care and chronic disease management services for many of their patients: “*The deferral of care for people who have chronic conditions or have prevention things that need to happen, it’s all been deferred and I’m anxious about that*” [NS1], with no clear means of addressing the backlog once the pandemic started to ease: “*Preventative care is definitely being de-prioritized and lost … so now we have a backlog … and honestly we haven’t figured out how we’re going to do that yet*” [ON4]. Participants also expressed frustration that their patients were not unable to access other necessary care in a timely manner: “*people aren’t getting tests in a timely fashion or aren’t seeing specialists in a timely fashion*” [NS6]. In many cases, FPs were unable to access the diagnostic tests and specialist care that their patients needed:

 “*We couldn’t access our diagnostic imaging. We couldn’t access our lab.… We require those services for us to work. And when we identify a problem and we need someone to have surgery, for example, I know they need it, but I can’t get a surgeon to see them because they’re not running any clinics.…” *[NS15].

 They expressed frustration at their inability to alleviate their patients’ suffering:

 “*…the frustration with waiting to get to see specialists and trying to manage these conditions that are clearly surgical without having surgical skills.… I saw a patient with almost complete blindness, … because of a cataract, which is immensely treatable. She’s now been waiting for a year and a half to get this treated and still has no date for her surgery*” [ON4].

 Participants described being unable to provide needed care to patients in crisis because of pandemic restrictions that limited unscheduled in-person care (ie, walk-in visits): “*the number of people that would drop in for, in crisis and needing various types of support, we weren’t really able to offer that*” [BC5]. The surge in patients presenting with mental health concerns had negative impacts on FPs’ own well-being and ability to empathize with their patients:

 “*[There was] a huge uptick in patients calling with anxiety and mental health complaints, which can be emotionally draining when you’ve talked to ten patients in a row about their anxiety and their depression. Those are heavy conversations, and they get tiring and you get compassion fatigue” *[NS21].

 Participants were particularly affected by the impact that pandemic policies and restrictions had on their ability to provide quality end-of-life care. Participants noted the contention between their desire to provide optimal patient care and the risk of infecting patients, providers, and family members with COVID-19. For example, a participant noted the personal risks associated with providing compassionate, patient-centred care in patients’ homes early in the pandemic, when personal protective equipment (PPE) was not widely available to community-based physicians:

 “*Everyone knew I was working with no PPE … the most heart-breaking piece for me was the palliative care piece because in the beginning, these patients had one choice – go to palliative care [in hospital] and die alone. That was the only thing that was being offered, and that’s not acceptable to the patient or the family. So the only way to [die at home] was through community health nursing and the [general practitioner], because the community health nurse needs the [general practitioner] to prescribe medications and we need to assess patients”* [NL11].

 Along with the personal risks of caring for patients in their homes during a pandemic, another concern was the availability of staff. A participant in Ontario noted that the redeployment of primary care nurses to other areas of the healthcare system (to assist with the pandemic response), compromised the FPs’ ability to provide palliative care:

*“Our palliative care team has never ever had a problem with nursing shortages, … Now we’re at a point where our primary care providers’ or palliative care providers’ capacity to care for patients in the community is completely hindered by the fact that we don’t actually have front-line nurses. Like no boots on the ground to help us out with that”* [ON5].

 Assuming additional responsibilities, such as taking over the tasks previously managed by nursing staff not only increased FPs’ workload, but also required them to carry out tasks for which they were not trained.

###  System-Related Stressors and Buffers of Burnout 

 Participants described four factors that, depending on their configuration, could either stress or buffer feelings of burnout: workload, payment model, locum coverage, and team and peer support. These factors related to attributes of the profession, such as the organization and financing of FP work, and depending on the positive or negative nature of the attribute, could either magnify or alleviate FPs’ sense of burnout.

####  Workload

 Participants noted that the demanding workload during the pandemic contributed to feelings of fatigue, stress, and burnout: *“People are just really tired… and they’re really, you know, we’ve been working like, non-stop through this and seeing lots of suffering”* [BC13]. Participants also described how the pandemic also required them to develop and adapt protocols for infection prevention and control in their offices: “*We were working almost around the clock for over a month making new protocols …That was a lot of work. And it’s ongoing”* [NS2]. This included the rapid adoption of virtual care:* “If it’s an entirely phone day, it can be very exhausting and draining. And it’s not more efficient, and so I think that there’s maybe a misperception that you can get a whole lot done when you’re doing it virtually*” [NS8]. The redeployment of nurses away from primary care also contributed to higher workloads for FPs, as one participant expressed:

 “*We had a nurse that kind of works with us [who coordinated] palliative care and our home visit plan… She was pulled aside and was mandated to go work at the COVID assessment … we had to retake over all of our palliative care and our coordination....” *[ON7].

 Participants noted that the demanding workload was: “*not sustainable, and I worked really hard these last nine months on this and it’s just, I can’t keep doing it or I’m going to get burnt out*” [NL4]. The overwhelming workload took a toll on participants and their families:

 “*The pandemic has compounded a very stressful situation. And, you know, the need is – I would use the word overwhelming. And so, to know how to set appropriate boundaries is very difficult in terms of just time put into work. But also, the stress of this job unfortunately does carry home more often than I would like and negatively impact my role as a husband and father and friend”* [NS20].

####  Payment Model

 Despite the increased workload associated with the pandemic, many fee-for-service practices struggled to adjust to lower in-person patient volumes and uncertainty around the implementation of virtual care fee codes, especially during the first wave of the pandemic:

 “*But just hearing from my colleagues that they had to lay off their receptionists, they were having trouble covering their rent and covering overhead, just because they weren’t seeing the number of patients they normally would. And I think there was a bit of delay with getting the virtual care codes covered. So, they were making phone calls and not getting paid for them, or not knowing if they’d ever get paid, but they still had that responsibility for their patients. And then just the … cost of PPE on top of all that was, it was really difficult for a lot of physicians” *[NL4].

 The worry of keeping financially afloat, coupled with the sense of duty to care for patients, greatly impacted fee-for-service FPs in the early weeks of the pandemic. A participant noted that fee-for-service contracts did not take pandemic considerations into account: “*The physician services agreement that we have with the Ontario government does not include pandemic care. There is no provision in that for what happens if my office gets shut down or if my expenses all of a sudden go up 50% or if my volume goes down 50%…” *[ON10]. Another fee-for-service participant in a solo practice described being unable to retain staff, eroding their capacity to manage the practice: “*I tried to keep my MOA [Medical Office Assistant] employed, but after a while it was just impossible. So, I had to lay her off … She’s an excellent MOA, but I can’t even afford to pay her now. And so, I am literally hanging on with my fingernails*” [BC10]. In contrast, participants whose remuneration model was based on per capita funding for rostered patients faced less financial uncertainty: “*Fortunately for me it was a family health organization, it didn’t impact my income a lot”* [ON18].

 Another participant described how Ministry payment rules, specifically those relating to the newly implemented fee codes for virtual care, made it challenging for her to provide care in a way that balanced the needs of patients with her own need to maintain a healthy work-life balance. She described her dilemma in trying to provide care over the Easter weekend (which, in most parts of Canada, includes a statutory holiday on Good Friday, along with routine office closures on the Saturday and Sunday):

 “*…the government says that you’re only allowed to talk to 40 people virtually. … last week I had 70 [patients] on Thursday that I have to call. … And Friday was a holiday, Saturday and Sunday, we’re not working … I went on Good Friday to the office alone and I called the rest of them, because I didn’t have heart to make them wait and I was doing everything I can do by the [rules]”* [NL12].

 Payment models also had an impact on participants’ well-being by affecting their ability to take time away from work. A FP whose practice was funded by an alternate payment plan (ie, non-fee-for-service) noted that the payment model allowed her to take much-needed breaks without worrying about lost income, which was in stark contrast to colleagues paid through fee-for-service:

 “*I can tell when I’m burning out, and I take time off. One of the nice things about being an APP [Alternative Payment Plan] physician is we have built in vacation. So, I can take a day off work. Or if I recognize what I really need is just a break, I can take a day or two off, and I still get paid. Which takes a heck of a lot of stress out of it for me. People in fee-for-service don’t have that same luxury” *[NS22].

 Participants also noted that the absence of paid sick leave was a significant financial concern, considering the heightened risk of exposure to COVID-19 and the subsequent consequences, including the possibility of becoming infected and having to self-isolate: “*What happens if you get sick? If I get sick, I’m off for a minimum of 14 days, but … I don’t qualify for any disability insurance or anything in that length of time or any other appropriate compensation*” [ON10]. A participant described the difficulties his father (who is also a physician) encountered when he was ill with COVID-19:

 “*The person [from our practice] who got COVID was my father, who’s a doctor, and he was in ICU and almost died… He was one of the original doctors providing a source of income and rent [for the practice]. When he was [in the hospital], he couldn’t even get worker’s compensation for payment, even though he acquired the COVID infection from a patient … at the clinic” *[BC9].

 The participant noted that despite the fact that the illness was acquired through work, his father was not covered by the provincial insurance program responsible for loss of income resulting from work-related incidents.

####  Practice Coverage and Access to Locums

 The inability to ensure patients have had access to services during their absence further contributed to FPs’ feelings of burnout. Furthermore, participants felt a sense of commitment and responsibility to their patients, which, despite their workload or feelings of burnout, compelled them to continue working. However, this sense of obligation also led to overextension, while at the same time reinforcing feelings of moral distress and burnout. A participant who worked with people who use substances noted that patients had urgent needs that would otherwise go unaddressed due to staffing shortages compounded by the pandemic:

 “*I’ve been on the brink of burnout for quite a number of months now [but] … if I didn’t do this work, we’re so short-staffed, there’s no one to step in, the patients are highly vulnerable. I’m sure you know this, but the overdose death rates absolutely sky-rocketed after COVID and so the urgency with wanting to try to make sure that people aren’t being lost to care, just became all that more important” *[BC5].

 Participants also felt a sense of responsibility to protect the functioning of emergency rooms and hospitals during the pandemic. A participant described the competing responsibilities of providing care while also trying to preserve emergency room capacity:

 “*I still have sick patients, I have well babies, I have post- partums, I have palliative patients, so it was kind of like, I know that that care can’t stop. … I couldn’t abandon my patients and I also couldn’t tell everybody to go to [the emergency department] with all their concerns because I work at [the emergency department] and they were doing everything they could to protect that resource”* [NL11].

 Participants struggled with choosing to take time off, as they recognized that their absence would result in longer wait times for appointments and potentially greater levels of patient frustration:

 “*If I take time off, it delays… it pushes back how long it takes to get in to book with me. I have to like come to grips with the guilt of ‘I’m putting my needs first’ so that I can then provide good care to my patients because I’m burning out. But then they’re frustrated that they can’t get in to see me in what they think is a reasonable amount of time. So, you can’t win”* [NS22].

 Participants also felt pressure to continue working due to the lack of locum coverage: “*I think a lot of the clinicians are getting burnt out. I’ve had a lot of people come to me and say, ‘I need to take a week off.’ And the locum supply is not where it should be, … [we need] a coordinated approach for locum coverage*” [BC9]. For participants, addressing patient needs was paramount, and routinely trumped their own need for taking time away from work.

####  Team and Peer Support

 Participants described how social isolation brought on by the pandemic negatively impacted their well-being: “*I’m the only doctor who consistently comes every day of the week… and the comradery that you used to have with the lunches and the walking through the hallway asking questions is gone now*” [BC9]. Peer support was consistently identified as a helpful support to combat loneliness as well as other sources of burnout. Participants noted the positive impacts of being part of a supportive team, who had shared experiences and could commiserate and encourage each other: “*I think that maybe my, being in a big team was really helpful for me… I really appreciated having the support with my team members there too*” [BC3] and “*I have a really great group of colleagues that I work with, both nurses and physicians. …we would all lean on each other during those times*” [NS16]. In each region, participants described meetings where colleagues could vent their frustrations and check-in on each other:“*We started doing Zoom meetings every week for an hour and that really, really helped, just talking with other people, checking in, and seeing how they were coping with everything*” [NL4]. Participants highlighted these meetings as a positive development to come out of the pandemic, and an initiative that many wanted to continue, even as some of the difficult work conditions eased:

 “*We started doing what we called weekly check-ins through Zoom. So, bring in all the physicians, bring in all the nurses and just let people vent for about an hour … you have no idea how much that improved the people’s demeanour. … and believe it or not, my colleagues don’t want me to stop these rounds, now they want every two weeks… it’s just that value of being connected to your peers*” [ON5].

## Discussion

 Throughout the interviews, FPs consistently expressed feelings of burnout, moral distress, and decreased professional and personal well-being. FPs described how the circumstances brought on by the COVID-19 pandemic impacted their ability to provide appropriate care for their patients, as they were often impeded by guidelines that prioritized patient and physician safety, and care for patients with COVID-19.^38^ As a result of these shifts in care provision, FPs were forced to reduce their delivery of routine or preventative care, resulting in concerns over patient outcomes and increased moral distress for FPs.^39^ Through various waves of the COVID-19 pandemic, FPs had to adapt their practices to accommodate severe shortages of PPE and other resources,^40,41^ the rapid transition to virtual care,^42-44^ variable patient volumes,^13,42^ and growing financial pressures (particularly for physicians practicing in fee-for-service models).^45^ The experiences described by participants in our study are reflective of those reported in similar previous studies examining FPs’ experiences of stress and burnout during the COVID-19 pandemic internationally.^12,46-51^

 We identified four interconnected factors that could magnify or alleviate the potential for burnout among FPs: workload, payment model, locum coverage, and team and peer support. Working in a team could can help alleviate a heavy workload and facilitate time away from work, without abandoning professional responsibilities. Conversely, fee-for-service payment requirements can increase workload and reduce the ability to take time away from work. These study findings highlight potential system-based interventions to address FP burnout, such as expansion of interprofessional team-based models of care, group practices, and alternate forms of FP payment models (ie, non-fee-for-service). In Canada, many of these interventions have been introduced through primary care reforms aimed at improving the quality of, or access to care^30^; however, our findings suggest that these may also promote FP well-being and alleviate burnout by allowing FPs to take time away from work, share workload, and gain collegial support, while highlighting the need for additional system-level reforms to address burnout specifically. A recent (February 2023) agreement in principle between the federal (Canada) and provincial governments to improve healthcare services, including a commitment to invest in teams and the health workforce, signals a growing recognition of the need for reforms that prioritize provider well-being.^52,53^

 Beyond these primary care reforms, our findings call for programs to provide short-term disability and illness insurance to enable FPs, particularly those practicing in fee-for-service practice models, to take sick leave and have access to funding to cover the fixed overhead costs of operating a practice. Currently, FPs in Canada have access to voluntary critical illness and disability insurance that covers their own income after a minimum absence (ranging from 30 to 90 days) but would not generally apply to shorter periods or isolation requirements introduced as part of public health guidelines during the pandemic.^54^ FPs also need access to organized locum tenens programs to ensure coverage of practices. System-level interventions identified in previous studies that examined physician burnout include increasing physician income, increasing physician numbers, and providing better practice opportunities for early-career physicians.^19^ In a survey of FPs in British Columbia, Hedden et al found that respondents favoured primary care reforms that supported direct employment, such as direct clinic funding, vacation and parental leave as opposed to policies tailored towards FPs continuing to operate as independent small business owners.^14^

 Additionally, during the pandemic, three provinces in our study (Ontario, Newfoundland and Labrador, and Nova Scotia) introduced remuneration policies to stabilize funding for fee-for-service practices that experienced cash flow issues due to sudden decrease in the volume of in-person visits and fixed overhead costs.^55^ In Ontario, fee-for-service physicians could access cash advances (to be paid back in future instalments) while Nova Scotia and Newfoundland and Labrador linked funding to the continued delivery of routine clinical care as well as specific COVID-19 related activities.^55-57^ Further research is needed to evaluate these programs, particularly their impact on physician burnout, decisions to close practices temporarily or permanently for the long term, and physician retirements.

 To date, interventions to combat FP burnout have largely targeted individual or unit-level organizational characteristics. Individual-level interventions that have been introduced to combat burnout include enhanced training in communication, stress management, and personal coping strategies, including building resilience, self-care, and spiritual health.^15,23-26,58,59^ Organizational-level interventions include reducing workload, increasing autonomy, improving team functioning, providing funding for administrative and clerical support, changing performance evaluation criteria, engaging physicians in leadership roles, and increasing employee involvement in pandemic response planning.^9,18,23,26,60-62^ Systematic reviews and meta-analyses have reported mixed results and call for more high-quality research evaluating the effectiveness of individual and organizational-level interventions, especially over the long-term.^9,63-66^

 Findings from this study echo calls for better system-level planning to alleviate workload issues related to the provision of care during a pandemic, such as increased involvement of primary care leadership and greater access to PPE.^26,28,41,43,67-71^ These findings, together with those from our larger project, indicate that poor system-wide planning contributed to the high levels of moral distress and burnout reported by FPs during the COVID-19 pandemic.

###  Limitations

 Interviews were completed between October 2020 and June 2021 in four Canadian provinces. Our findings may not reflect the experiences of FPs in other areas or at later stages of the COVID-19 pandemic. Although we used maximum variation sampling and a variety of recruitment approaches, we may not have fully captured some perspectives. For example, most FPs in Canada are paid by fee-for-service, while the majority of our participants were paid by alternate payment plans. Similarly, the majority of our participants were women, and men’s experiences may not be fully represented. We also did not define burnout or moral distress and injury in the interview, but relied instead on participants’ self-report. Additionally, all participants were clinically active at the time of the interview; future research should examine the perspectives of FPs who are diagnosed with burnout and/or moral injury using validated tools, as well as the perspective of FPs who left the workforce as a result of burnout.

## Conclusion

 Although burnout and moral distress experienced by FPs have been documented for a long time, the COVID-19 pandemic amplified FPs’ inability to provide appropriate care and contributed to moral distress and burnout. FP burnout was attributed to a series of overlapping, interconnected stressors and buffers: workload, payment model, locum coverage, and team and peer support. System-wide interventions to mitigate stressors and strengthen buffers include the expansion of interprofessional team-based models of care and group practices, alternate forms of FP payment models (ie, non-fee-for-service), organized locum programs, and the availability of short-term insurance to cover fixed practice operating costs. Better planning to address sources of moral distress and burnout are also needed to support FPs during pandemics and other public health emergencies that disrupt the routine provision of primary care and other health services.

## Ethical issues

 We obtained approval from the research ethics boards at Simon Fraser University and the University of British Columbia (through the harmonised research ethics platform provided by Research Ethics British Columbia), the Health Research Ethics Board of Newfoundland and Labrador, Nova Scotia Health, and Western University. Participants provided informed consent before interviews were scheduled. All methods in this study were performed in accordance with the relevant ethical guidelines and regulations.

## Competing interests

 Authors declare that they have no competing interests.
